# Estimating tuberculosis incidence from primary survey data: a mathematical modeling approach

**DOI:** 10.5588/ijtld.16.0182

**Published:** 2017-04-01

**Authors:** S. Pandey, V. K. Chadha, R. Laxminarayan, N. Arinaminpathy

**Affiliations:** * Public Health Foundation of India, New Delhi; † Epidemiology and Research Division, National Tuberculosis Institute, Bangalore, India; ‡ Center for Disease Dynamics, Economics & Policy, Washington, DC; § Princeton Environmental Institute, Princeton, New Jersey, USA; ¶ Department of Infectious Disease Epidemiology, Imperial College London, London, UK

**Keywords:** ARTI, prevalence, transmission, duration

## Abstract

**BACKGROUND:**

There is an urgent need for improved estimations of the burden of tuberculosis (TB).

**OBJECTIVE:**

To develop a new quantitative method based on mathematical modelling, and to demonstrate its application to TB in India.

**DESIGN:**

We developed a simple model of TB transmission dynamics to estimate the annual incidence of TB disease from the annual risk of tuberculous infection and prevalence of smear-positive TB. We first compared model estimates for annual infections per smear-positive TB case using previous empirical estimates from China, Korea and the Philippines. We then applied the model to estimate TB incidence in India, stratified by urban and rural settings.

**RESULTS:**

Study model estimates show agreement with previous empirical estimates. Applied to India, the model suggests an annual incidence of smear-positive TB of 89.8 per 100 000 population (95%CI 56.8–156.3). Results show differences in urban and rural TB: while an urban TB case infects more individuals per year, a rural TB case remains infectious for appreciably longer, suggesting the need for interventions tailored to these different settings.

**CONCLUSIONS:**

Simple models of TB transmission, in conjunction with necessary data, can offer approaches to burden estimation that complement those currently being used.

## RESUME

**CADRE:**

Il y a un besoin pressant d'améliorer l'estimation du poids de la tuberculose (TB).

**OBJECTIF:**

Elaborer une nouvelle méthode quantitative basée sur un modèle mathématique et de démontrer son application à la TB en Inde.

**SCHEMA:**

Nous avons élaboré une modèle simple de la dynamique de transmission de la TB, afin d'estimer l'incidence annuelle de la TB maladie à partir du risque annuel d'infection tuberculeuse et de la prévalence de TB à frottis positif. Nous avons d'abord comparé les estimations des modèles pour les infections annuelles par cas de TB à frottis positif, en utilisant des estimations empiriques préalables venant de Chine, de Corée et des Philippines. Nous avons ensuite appliqué le modèle afin d'estimer l'incidence de la TB en Inde, stratifiée par zone urbaine et rurale.

**RÉSULTATS:**

Les estimations des modèles sont en accord avec les estimations empiriques préalables. Appliqué à l'Inde, le modèle suggère une incidence annuelle de TB à frottis positif de 89,8 par 100 000 habitants (IC95% 56,8–156,3). Les résultats mettent également en évidence un contraste entre TB en zone urbaine et rurale : si un cas de TB en zone urbaine infecte davantage d'individus par année, un cas de TB en zone rurale reste contagieux nettement plus longtemps, ce qui suggère un besoin d'interventions spécifiques aux différents contextes.

**CONCLUSION:**

Des modèles simples de transmission de la TB, en conjonction avec les données nécessaires, peuvent offrir des approches à l'estimation du fardeau qui sont complémentaires à celles actuellement en usage.

## RESUMEN

**MARCO DE REFERENCIA:**

Existe una necesidad urgente de mejorar las estimaciones de la carga de morbilidad por tuberculosis (TB).

**OBJETIVO:**

Elaborar nuevos métodos cuantitativos fundamentados en la modelización matemática y describir su aplicación a la TB en la India.

**METODOS:**

Se construyó un modelo sencillo de la dinámica de transmisión de la TB, con el objeto de calcular la incidencia anual de la enfermedad, a partir del riesgo anual de contraer la infección tuberculosa y la prevalencia de TB con baciloscopia positiva. En primer lugar, se compararon las estimaciones del modelo de las infecciones anuales por casos de TB con baciloscopia positiva, aplicando estimaciones empíricas anteriores de la China, Corea y las Filipinas. Luego, se aplicó el modelo con el fin de estimar la incidencia de TB en la India, estratificada por los entornos urbano y rural.

**RESULTADOS:**

Las estimaciones del modelo fueron concordantes con las estimaciones empíricas anteriores. Al aplicarlo a la India, el modelo indicó una incidencia anual de TB con baciloscopia positiva de 89,8 por 100 000 habitantes (IC95% de 56,8 a 156,3). Los datos también pusieron en evidencia diferencias en la TB urbana y rural, pues un caso de TB en medio urbano contagia más personas por año, pero un caso en entorno rural permanece contagioso durante un período más prolongado. Estos resultados destacan la necesidad de intervenciones específicas en cada entorno.

**CONCLUSION:**

Los modelos sencillos de transmisión de la TB, aunados a los datos necesarios, ofrecen estrategias de estimación de la carga de morbilidad que son complementarias de los enfoques utilizados en la actualidad.

TUBERCULOSIS (TB) is a major global public health challenge. Of the estimated 10.4 million cases globally in 2015, only about three fifths were notified to the public health authorities.^1^ The ‘missing cases’ pose a serious challenge to TB control. To design appropriate case-finding interventions and gauge the efficiency of the public health system in capturing TB cases, it is imperative to have more precise estimates of TB incidence, especially in high-burden countries such as India.^2^ However, in any given national setting, estimating annual TB incidence is challenging. The direct measurement of active TB incidence requires large study populations to be followed for a year or longer, while carefully accounting for TB cases entering or leaving the population during this time. The resources, personnel and funding needed to sustain this effort in high-burden, low-income countries render such direct measurements impractical.^3^

An alternative approach using the annual risk of tuberculous infection (ARTI, i.e., the proportion of non-infected individuals who acquire infection each year) was proposed by K Styblo in the 1980s.^4^ Taking into account the estimates available at the time (each smear-positive case of TB is infectious for a duration of 2 years on average before cure or death, and causes on average 10–12 infections per year during this time), Styblo estimated that an ARTI of 1% corresponded to 50 incident smear-positive pulmonary TB cases a year. This allowed a rough estimation of incidence to be projected from ARTI rates, which were in turn estimated from prevalence of infection surveys. This parametric relationship was observed primarily in the pre-chemotherapy period, and is becoming increasingly outdated in the present era, where DOTS implementation has profoundly altered TB epidemiology. Recent work illustrates how, in China, the Philippines and the Republic of Korea, estimates for annual infections per case derived from the available data range from 2.6 to 5.8, consistently lower than those used by Styblo.^4^ Earlier modelling work reported a range of 3.8 to 7.9 in these countries,^5^ with estimates in other countries varying more widely. In the light of these issues, incidence estimation today is based increasingly on notifications of cases from routine surveillance, together with estimates of the extent of underreporting and underdetection. The latter are drawn from expert opinion, although inventory studies are increasingly being used to obtain information about underreporting.^6^

In the present study, we present a complementary approach for estimating TB incidence. Our approach relies on a simple dynamic model of TB transmission, designed to estimate the key transmission parameters (annual number of infections and duration of infectiousness) from primary data available from surveys to measure the prevalence of infection and active TB disease. From these estimates, the model projects the annual incidence of TB disease, independently of assumptions about the extent of underreporting (and underdetection). We apply this framework to estimate the TB burden in India, estimated to account for 25% of the global TB burden. While nationally representative surveys to estimate the ARTI have been carried out in India, there are no nationally representative surveys of TB disease prevalence. However, various recent surveys at the subnational level suggest marked differences between urban and rural settings, as discussed below. We therefore separately applied the model to prevalence estimates from rural and urban settings. In addition to estimating the TB burden, our approach sheds light on the possible reasons for the differing TB epidemiology in urban and rural settings in India.

In this paper, we describe the model framework, its structure and how it relates to data from infection surveys. We first check consistency between model findings and independent estimates reported by van Leth et al. We next apply the study model to estimate the TB burden in India, and determine the model inputs that are most important for improving the precision of incidence estimates. Finally, we discuss some limitations of the approach, and outline ways in which the basic model framework can be developed, refined and validated in future.

## METHODS

**Figure 1.**
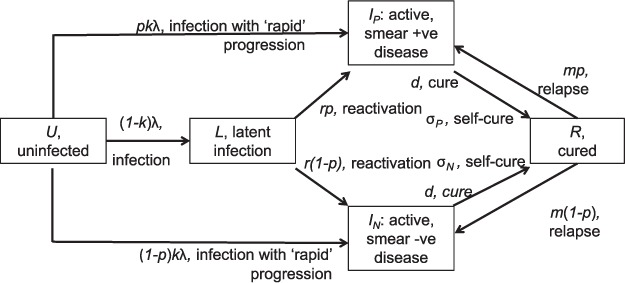
Schematic illustration of the model structure. The population is divided into different compartments, with flows between compartments given by terms on arrows (terms identified in Table 1). Model equations are given in the Methods; symbols are as follows: *p*, proportion of cases smear-positive; *k*, proportion of infections being ‘rapid’ progressors; λ, annual risk of TB infection; *c*, infectiousness of smear-negative TB relative to smear-positive; *r*, rate of progression from remote infection to active disease per year; *m*, per-capita rate of relapse to active disease; *d*, per-capita rate of initiation on curative therapy; *σ*, per-capita rate of spontaneous cure. Smear +ve = smear-positive; smear +ve = smear-negative.

We used a deterministic, compartmental model of TB transmission dynamics (Figure 1). The model presented here is the simplest possible framework necessary to fit the available data; for the sake of simplicity, we ignored age structure, as well as the acquisition and transmission of multidrug-resistant TB. While the rate of diagnosis and cure can differ by health care sector (public vs. private), we assumed rates that were averaged across these settings. However, a necessary feature of the model is to distinguish smear-positive from smear-negative TB, to reflect the stratification by smear status typically supplied by survey data.

Briefly, in the model the population is divided into different categories: uninfected (*U*), latent infection (*L*), active disease (distinguishing smear-negative [*I*_N_] from smear-positive disease [*I*_P_]) and cured (*R*). Population flows between these states are represented by the following system of ordinary differential equations that capture TB transmission dynamics as well as other factors, including breakdown to active disease, mortality, cure and relapse.

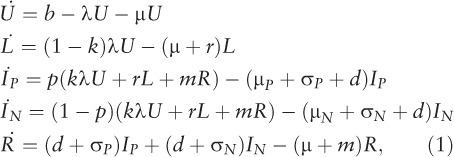
where *b* is the per-capita birth rate, *k* is the proportion of infections progressing ‘rapidly’ to active disease, *r* is the per-capita rate of breakdown to active disease, *m* is the per-capita rate of relapse to active disease, μ is the background mortality rate, μ*_P_* and μ*_N_* are the per-capita mortality rates for smear-positive and smear-negative cases, respectively, and σ*_P_* and σ*_N_* are the per-capita rates of self-cure.

The force-of-infection λ is given by:

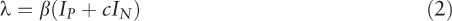
where *c* denotes the diminished transmission potential of smear-negative cases relative to smear-positive cases.

This leaves two parameters to be estimated: *d*, the per-capita rate of diagnosis and cure through treatment; and β, the average number of infections arising per year per smear-positive case. We note here that the average duration of disease *D* arises from a combination of mortality, spontaneous cure and cure through diagnosis and treatment, i.e.:

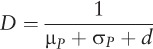
for smear-positive cases, and likewise for smear-negative cases. In the Styblo framework, β was estimated at 10 infections, while *D* was estimated at 2 years. Here we calibrate these to the available data, as described below. Although we estimated the rate *d*, we present results for the overall duration of disease *D* using the equation above, as this is a quantity that is more readily understood for the purpose of TB epidemiology. For the sake of simplicity, we assumed the same rate of diagnosis and treatment for smear-positive as for smear-negative TB (*d*)—an assumption that can be relaxed if there are more quantitative data to inform these relative rates.

In a given setting, key data inputs for the model are ARTI (equivalent to λ in equation [2]) and the prevalence of smear-positive TB (equivalent to *I*_p_). We describe the sources of these parameters: first, ARTI is a measure of the force of infection, defined as the probability of acquiring new tuberculous infection or re-infection over a period of 1 year, and is derived mathematically from the prevalence of infection estimated by tuberculin surveys. The ARTI values used in the present study were derived by pooling the cluster survey data from four zonal level surveys conducted among children aged 1–9 years from 2009 to 2010.^7^

Second, prevalence of TB disease is defined as the proportion of people suffering from TB disease at a given point of time. Prevalence values used in the present study were pooled estimates obtained from nine subnational disease prevalence cluster surveys conducted in India during 2006–2012.^8–15^ In these surveys, representative samples of individuals (age ⩾15 years) in the respective areas were screened using interviews for the presence of symptoms suggestive of pulmonary TB and/or by chest radiography (CXR) using mass miniature radiography and digital radiography at one site. Those found to have symptoms and/or any radiological abnormality on CXR underwent smear sputum examination (two specimens), as well as culture using solid media. Screening by both interview and CXR was undertaken at five of these sites, while screening by interview only was used at four other sites. Prevalence was estimated after correcting for the bias introduced due to incomplete data using logistic regression model with robust standard error and missing value imputation. Prevalence estimates for the sites where screening was conducted by interview only were corrected for non-screening by radiography using the correction factor obtained from sites where both screening tools were used.

For the purpose of the present study, we obtained the national level weighted estimates for prevalence of smear-positive pulmonary TB by pooling the estimated prevalence at the nine sites individually, the weights being equal to the inverse of variance and corrected for paediatric age group; it was assumed that 9% of cases occurred among children^16^ and 30% of the population belonged to the paediatric age group.^17^ While these prevalence estimates represented the best estimates for the country, they were limited by the fact that the survey sites had not been selected to be representative of the country. However, the ARTI estimates were nationally representative.

Natural history parameter inputs for equation (1) were drawn from the literature (Table 1). The per-capita rates of TB mortality and self-cure in equation (2) were inferred from a recent systematic review (Appendix).^23^^*^ Solving the model at equilibrium, we were able to determine the values of β and *d* necessary to yield the correct ARTI and smear-positive prevalence. After determining these values, the annual incidence can be calculated mathematically using the equation given in the Appendix.

**Table 1:**
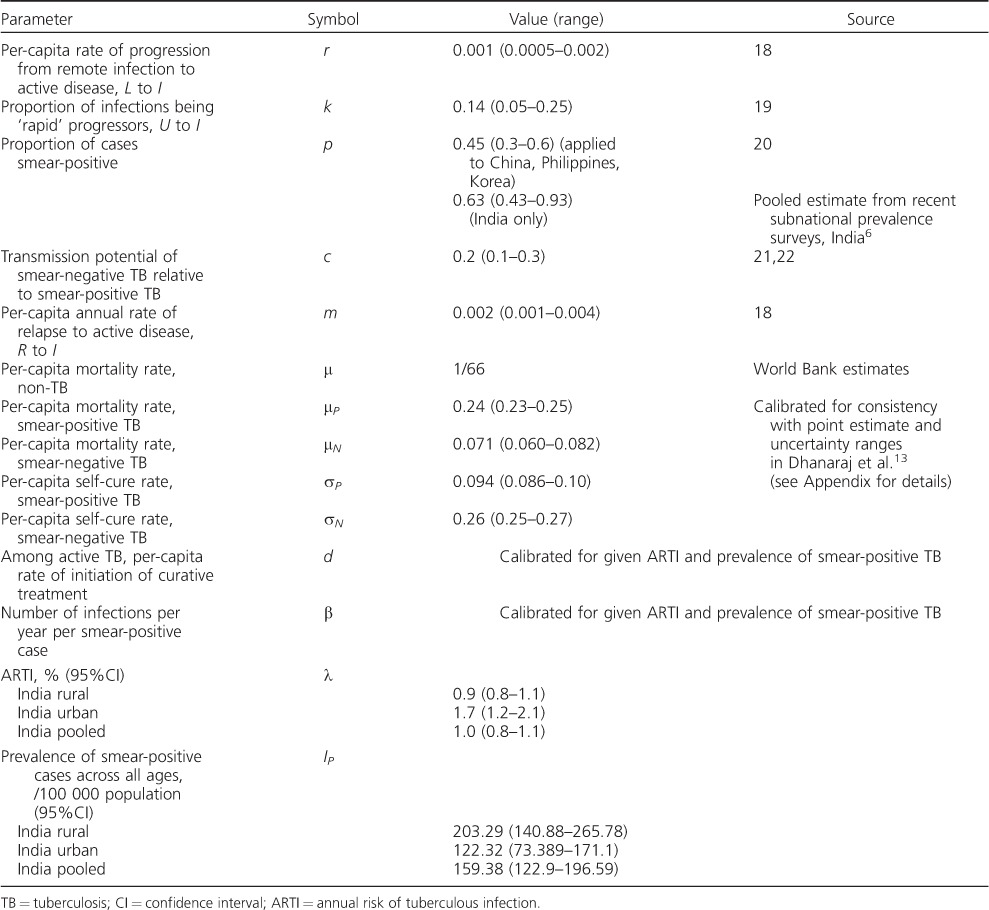
Summary of input parameters for the model

To appropriately propagate the uncertainty from inputs to incidence estimates, we determined log-normal distributions for each parameter (as well as ARTI and prevalence) to capture the uncertainty ranges (Table 1). Taking the lower bound, point estimate and upper bound as the 2.5th, 50th and 97.5th percentiles, respectively, we chose the mean and variance of a log-normal distribution for each input parameter to capture these percentiles using least-square estimations. Taking 100 000 independent samples for each of these inputs, we then recorded the estimated incidence for each sample. From the resulting set of 100 000 outputs, we calculated the point estimates and 95% credible intervals as the median, 2.5th and 97.5th percentiles, respectively. A computationally efficient method for conducting these 100 000 iterations is described in the Appendix.

**Figure 2.**
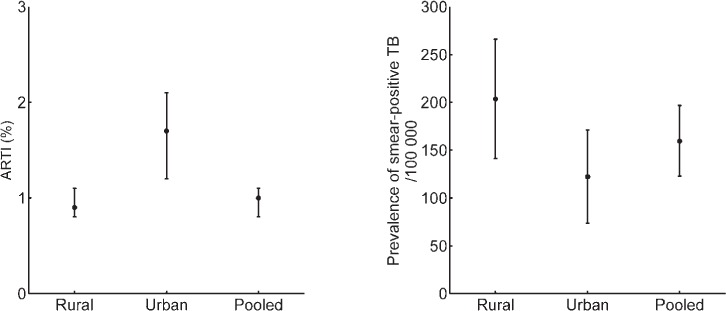
Results of pooled prevalence surveys, showing contrasting epidemiology between urban and rural tuberculosis in India. ARTI = annual risk of tuberculous infection.

We applied this model in the following way: first, as a consistency check, we compared our model findings for β against those derived by van Leth et al. from successive prevalence surveys in three different country settings—China, the Republic of Korea and the Philippines—independently of Styblo's estimates of 10–12 infections per prevalent case per year. We then applied the method for the estimation of TB incidence in India. Figure 2 shows findings from subnational prevalence surveys across the country (see Table 1 for data). It should be noted that although urban areas indicate higher ARTIs than in rural areas, the prevalence of smear-positive pulmonary TB is higher in rural areas. We therefore fitted the model separately to ARTI and prevalence inputs consistent with ‘urban’ and ‘rural’ TB to reflect the difference in TB epidemiology in these settings.

While most other parameter values were drawn from the general literature, here we used India-specific estimates for the proportion of smear-positive cases, again drawn by pooling the data from subnational prevalence surveys (unpublished data). Finally, to examine the sensitivity of the model to a given parameter, we explored the effect of fixing the parameter in question on the precision of model estimates for smear-positive TB incidence. In particular, we measured the interpercentile range in smear-positive incidence as the difference between the 2.5th and 97.5th percentiles. We first found this range for the ‘full’ model, where all parameters were allowed to vary simultaneously, as described above. By holding a given parameter fixed at its central value, we then re-estimated the incidence by varying the remaining parameters to record the resulting reduction in the interpercentile range. By repeating this for all model parameters, we were able to identify the most sensitive parameters as those associated with the greatest increase in precision (i.e., the greatest reduction in interpercentile range). We were thus able to determine which specific inputs would be most important to improve the precision of these incidence estimates.

No ethics approval was required, as the work presented here involved secondary analysis of data reported in earlier studies.

## RESULTS

**Figure 3.**
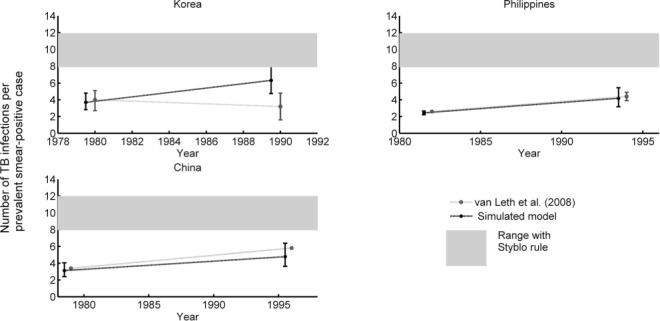
Comparison of estimates for the number of infections per smear-positive TB case per year. Estimates from Styblo (shaded bar), those derived from successive prevalence surveys in van Leth et al. (grey points), and those derived by the present model (black points) are shown. TB = tuberculosis.

Figure 3 compares model findings with results reported by van Leth et al. for β estimates in China, the Philippines and Korea.^4^ Shaded regions illustrate the parameter space corresponding to Styblo's rule: as noted in van Leth et al.,^4^ the assumption of 10–12 infections per year is consistently higher than independent estimates by van Leth et al. (points in grey). However, model estimates for β appear broadly consistent with these latter points (comparing estimates in grey vs. those in black). Model estimates for β were exceptionally higher for Korea in 1990; however, certain aspects of that survey, described below, may account for this discrepancy.

**Figure 4.**
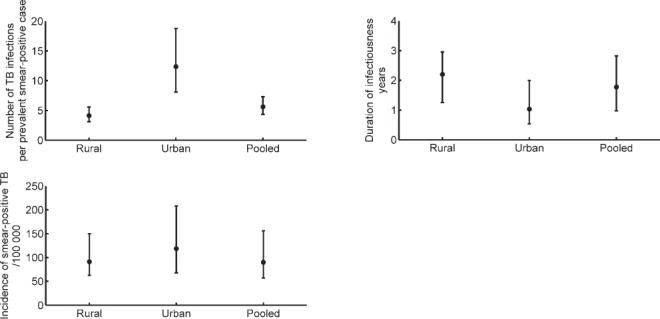
Application of the model to prevalence survey data from India. Owing to different TB epidemics in urban and rural settings, estimates for these settings are shown separately. TB = tuberculosis.

Applying the model to India, Figure 4 shows the results for β, *D* and incidence estimates in urban and rural settings, along with nationally pooled estimates (see also Table 2). The figure suggests that the annual number of infections per smear-positive case tends to be higher in urban than in rural areas, while the duration of infectiousness is the opposite. Overall, rural areas tend to have lower incidence than urban areas; however, a national-level estimate could obscure these heterogeneities.

**Table 2:**
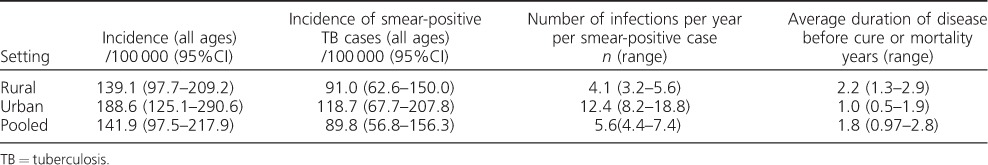
Summary of model output results: for rural, urban and pooled (rural + urban) settings in India

**Figure 5.**
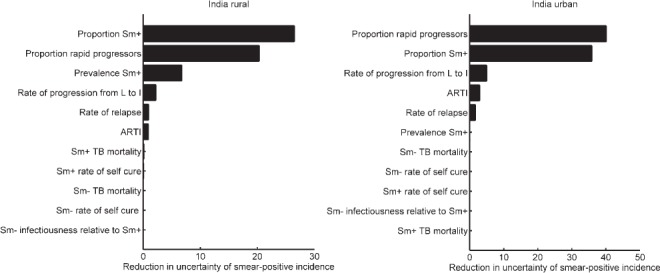
Sensitivity analysis with respect to smear-positive TB incidence. Bars show the reduction in the interpercentile range in Figure 4C (i.e., between the 2.5th and 97.5th percentiles of incidence estimates) when each parameter is, in turn, kept fixed at its central value (Table 1). Of all the model inputs, these results suggest that the precision of incidence estimates would be most improved by improved precision in *k*, the proportion of infections progressing ‘rapidly’ to active disease (within 2 years of infection), and *p*, the proportion of cases that are smear-positive. The remaining parameters are as shown in Table 1. ARTI = annual risk of tuberculous infection; TB = tuberculosis; Sm+ = smear-positive; Sm− = smear-negative.

**Figure A.2:**
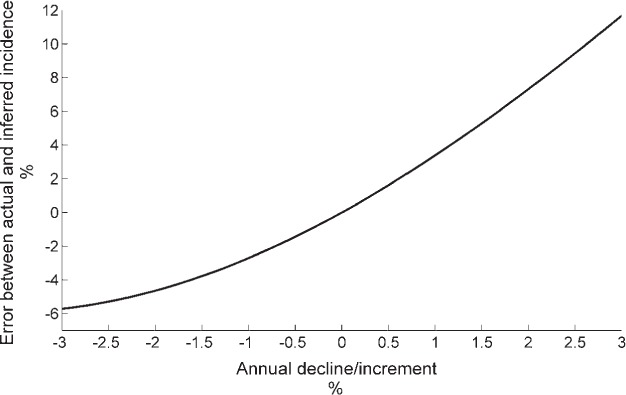
Bias in incidence estimates when an equilibrium model is applied to a changing epidemic.

**Figure A.3:**
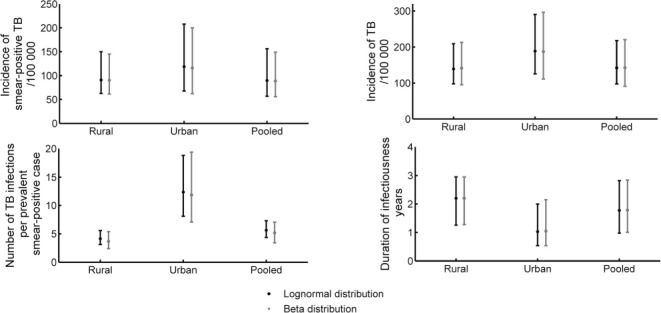
Comparison of model outputs when using β distributions for all input parameters (black) rather than log-normal distributions (grey). TB = tuberculosis.

The uncertainty intervals on these incidence estimates, while relatively wide, reflect a model-based aggregation of the uncertainty in input parameters. To address the role of individual parameters and inputs in model uncertainty, Figure 5 shows the parameter sensitivity, estimated (as described above) by keeping a given parameter fixed on the precision of model estimates for smear-positive incidence. The figure shows that, while the model is guided by ARTI and prevalence data, certain natural history parameters can nonetheless have greater impact on incidence estimates. The figure highlights three parameters in particular: the proportion of infections that are ‘fast’ progressors, the rate of progression from latent infection to active disease and the proportion of smear-positive cases. As discussed below, the relative role of these different inputs appears to differ according to setting (urban vs. rural). Appendix Figure A.2 also shows the potential bias in incidence estimates arising from the assumption of an equilibrium epidemic. In brief, this analysis shows that our approach tends to underestimate incidence if the ‘true’ underlying epidemic is a declining one, and vice versa. Appendix Figure A.3 shows the sensitivity of these results to the choice of distribution for input uncertainty (employing β distribution rather than log-normal distributions); estimates and uncertainty were not substantially altered.

## DISCUSSION

The estimation of TB incidence in high-burden settings is an important but difficult task. Even if incidence cannot be measured directly, it may nonetheless be reflected in other data that are more readily measured, for example through prevalence surveys. In this context, models of TB transmission can help us estimate the underlying incidence that best explains a given set of data. By circumventing the need to make a priori assumptions regarding the ratio of incidence to notifications or the duration of infectiousness, the model presented here complements methods currently used by the World Health Organization for estimating TB incidence.

This framework serves to show the potential value of ARTI and prevalence in estimating TB burden: these estimates should be regarded as a first step that can be refined and improved in future. For example, it is of note that certain natural history parameters can be more important in increasing the precision of incidence estimates than epidemiological inputs for ARTI and prevalence (Figure 5). This may be partially due to the relatively wide uncertainty intervals that we assumed for natural history parameters (Table 1). It should be noted, however, that two of these key parameters govern the lifetime risk of developing active disease from tuberculous infection—the proportion of ‘fast’ progressors and the rate of breakdown to active disease. These have previously been recognised as critical underlying parameters in the context of TB transmission dynamics.^24^ The third key parameter, the proportion of incident cases that are smear-positive, is clearly important in estimating the incidence of smear-positive TB. More setting-specific and precise estimates of these parameters would contribute to improved incidence estimates. Information on disease progression may require dedicated cohort studies, while data on smear-positive proportions would be more directly available from prevalence surveys (as we have used, for example, for India). In the future, more developed models incorporating additional sources of data such as TB mortality and patient care-seeking pathways, together with public sector notifications, could yield improved incidence estimates that are more comprehensively sourced from the available data.

It is also of note that the relative importance of ARTI and prevalence appears to vary according to setting, with prevalence being substantially more important than ARTI in determining the precision of incidence estimates in rural settings (Figure 5A), but with negligible impact in urban settings (Figure 5B), particularly if there is uncertainty regarding natural history parameters. These patterns could be understood in terms of our parameter estimates, which indicate that in rural settings, where β is generally lower (Figure 4B), transmission is driven by a large prevalent pool (Figure 2B), rather than by high infectiousness per case. In urban settings, the converse is true.

Where independent estimates for β are available, these are mostly consistent with model-based estimates calculated on the basis of prevalence and ARTI inputs (Figure 2). In the case of Korea in 1990, however, the model appears to show significantly higher estimates for β than those estimated independently. It is to be noted that ARTI estimates in that case were not as robust as in other years due to non-ascertainment of the mode of tuberculin reaction sizes that would represent true tuberculous infection.^25^ Moreover, TB control activities in the period shown may have led to a marked change in the proportion of TB cases that were smear-positive. While offering a possible reason for the difference between model-based and independent estimates of β, this also underscores the importance of the inputs used in ensuring the robustness of the model estimates.

**Figure A.4:**
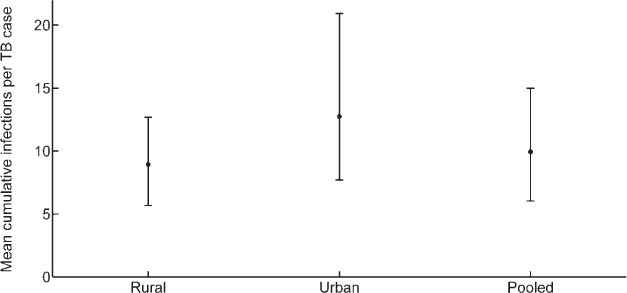
Total number of infections per TB case in each of the different settings presented in the main text. TB = tuberculosis.

Given the strikingly different nature of the TB epidemic in urban and rural settings in India (Figure 2), our approach has the added benefit of shedding light on potential reasons for such heterogeneity. A TB patient tends to transmit to more individuals in urban settings, but tends to have a longer duration of disease in rural settings (Figure 4). To our knowledge, this is the first time such potential drivers have been quantified. A higher population density in urban areas is consistent with the higher number of infections per year per smear-positive TB case, while limited access to health care could be one explanation for the longer duration of untreated TB in rural areas, which in turn could lead to the relatively high prevalence in these settings. Nonetheless, our results suggest that a TB case in an urban setting tends to cause more cumulative TB infections over the duration of an infectious period than an individual in a rural setting (Appendix Figure A.4).

Further work is needed to explore the mechanisms behind these findings. Nonetheless, the data (as well as our analysis) suggest the need for more urban- and rural-specific TB interventions in India. In particular, airborne infection control, including reduced crowding and improved ventilation, could play a more important role in urban TB control than in rural settings. Conversely, while timely diagnosis and treatment is crucial for controlling both urban and rural TB, our duration estimates suggest that they could have an especially pronounced impact in rural settings.

The methodology that we propose has some limitations. First, this approach neglects the role of human immunodeficiency virus (HIV) infection in TB transmission and is thus best suited in settings, as in much of India, where HIV-TB coinfection is low. Second, we have adopted an equilibrium model for simplicity. Such an approach is helpful in settings where prevalence data are only available for one point in time. Appendix Figure A.2 suggests that, where the underlying epidemic is slowly varying in time, any bias introduced by an equilibrium model is likely to be small. Nonetheless, the potential for repeat prevalence surveys to inform a non-equilibrium model is an important topic for future work. Third, caution should be used when assuming our ‘pooled’ estimates to represent national TB incidence in India: unlike ARTI data, the prevalence data used here are not necessarily nationally representative. Further research should address the validity—when seeking nationally representative estimates—of aggregating urban and rural data at the input stage, rather than modelling them separately and aggregating the model outputs. In this study, the former approach simply serves as a helpful indication of the ‘average’ epidemiological conditions nationwide.

A fourth limitation is in implementation: our work draws on data on both latent infection and disease. In practice, however, there has been a decreasing emphasis on tuberculin surveys to measure the prevalence of latent tuberculous infection due to the challenges in interpreting survey data, in particular, with declining rates of infection increasingly causing difficulties in distinguishing true tuberculous infections from cross-reactions. In future, newer tuberculins more specific to infection with Mycobacterium tuberculosis, or a more practicable method of collecting blood specimens among children in field conditions for interferon-gamma release assays, may help overcome this problem. Another justification for recent shifts away from infection surveys is that the identification and treatment of TB disease offer more immediate health gains than the diagnosis of individuals with latent infection, the majority of whom may not progress to active disease. Nonetheless, our work emphasizes the potential value of measuring latent infection for estimating the incidence of active disease.

Overall, our work highlights the need for improved estimates for TB burden at the subnational levels (for example, precise estimates by state, and by rural, urban, slum and tribal areas), as attention turns towards the millions of cases going undetected worldwide each year. Alongside the potential for future diagnostic tools and improved prevalence surveys, new analytical methods can offer informative and complementary approaches for the benefit of public health.

## Figures and Tables

**Figure A.1:**
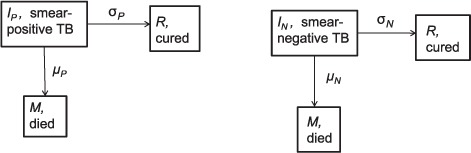
Schematic illustration of a simple cohort model (without transmission dynamics), tracking mortality and self-cure in smear-positive and smear-negative TB cases. TB = tuberculosis.

## Footnotes

^*^The appendix is available in the online version of this article, at http://www.ingentaconnect.com/content/iuatld/ijtld/2017/00000021/00000004/art00004

## APPENDIX

The model, specified in equation (1) in the main text and illustrated in Figure 1, was used to calculate the equilibrium state; model-simulated annual risk of tuberculous infection (ARTI) and prevalence were determined as follows:

The incidence of smear-positive TB at equilibrium is thus given by

that is, an integral over a unit time interval (1 year) of all influx terms into the state *I*_p_ in Figure 1.

### An efficient method for solving the system at equilibrium

For given values of β and *d*, equation (1) in the main text is solved to find the solution at equilibrium. Simulated values for ARTI and smear-positive prevalence are thus given by respectively λ and *I*_P_. Given data for ARTI and prevalence, we choose β and *d* to minimise the sum of least-squares between model-simulated and data values.

The simplest, most direct way to do this for a given β and *d* is to simulate an epidemic to equilibrium, determine ARTI and prevalence, and repeat to adjust β and *d* using a simplex algorithm. However, when incorporating uncertainty into the calculation, the process has to be repeated over 10 000 samples, and thus becomes very time-consuming. A more efficient approach is as follows.

At equilibrium, the derivatives in equation (1) are set to zero. Rather than calculating λ using (2), we treat λ and *I*_P_ as known (and given by the data), substituting this into the system of equation (1) to obtain a system of simultaneous equations in the four unknowns *U, L, I_N_* and *d*. Owing to the product of *d* and *I*_N_ in the fourth equation in (1), this remains a nonlinear system. Nonetheless, together with the constraint that *U+L+I_P_+I_N_+R=*1, this system can be easily and efficiently solved using the Newton-Raphson method.

### Estimating mortality and spontaneous cure rates

We draw from Dhanaraj et al.,^13^ a systematic review of tuberculosis (TB) outcomes in the pre-chemotherapy era. In brief, this study suggested that the mean duration of untreated TB is around three years. Smear-positive TB has a case-fatality rate of roughly 70%, while smear-negative TB has a case-fatality rate of roughly 20%. To capture the relationship between these outcomes and the per-capita hazard rates used in the present study, we used a simple ‘cohort’ model (Figure A.1).

Solving this simple model in the case of smear-positive TB with the initial conditions

*I_P_* = 1, *C_P_* = *M_P_* = 0, it is clear that, as a function of time:

A mean duration of 3 years thus implies that 1/(μ*_P_* + σ*_P_*) = 3, and a case fatality rate of 70% suggests that μ*_P_*_/_(μ*_P_* + σ*_P_*) = 0.7. Together, these imply that μ*_P_* = 0.23, σ*_P_* = 0.1. Similarly, for smear-negative TB, we have μ*_N_* = 0.067, σ*_N_*=0.27. We obtain uncertainty intervals for these rates by applying this procedure to the upper and lower bounds for the outcome estimates provided in Dhanaraj et al.^13^

### Assessing potential bias when applying an equilibrium model to a changing epidemic

We examined the potential bias arising from the simplifying assumption of an equilibrium epidemic by applying the model to simulated data, where incidence is changing at a given rate. In particular, the current World Health Organization approach to the TB burden in India assumes an epidemic that was at equilibrium until 2001, with a 1.5% decline in incidence thereafter arising from improving socioeconomic conditions.^1^ We simulated an epidemic at equilibrium until 2001, and in subsequent years subject to a given, annual change in β. Recording simulated data for ARTI and prevalence as of 2015 from this epidemic, we estimated the incidence in 2015 using the equilibrium model approach, ultimately to find the error in the incidence estimate. Figure A.2 shows the results of this analysis. For the purpose of exploration, we did not limit the trend in the simulated ‘true’ epidemic to declining β, but incorporated a range of scenarios from a 3% annual decline to a 3% annual increase. The figure suggests that, for a declining TB epidemic, the model tends to underestimate incidence, and *the opposite is true in the case of a growing TB epidemic*. Nonetheless, there remains good quantitative agreement between estimated and underlying TB incidence.

It is assumed that ‘true’ incidence (simulated, for the purpose of comparison) has been changing at a steady rate since 2001, the rate indicated by the *x*-axis. Given ARTI and prevalence in 2015, we then apply the incidence estimation method to estimate incidence in 2015, to compare this with the ‘true’ (simulated) incidence. Results illustrate that the model tends to underestimate incidence in the event of a steadily declining epidemic, and vice versa for a growing epidemic.

### Sensitivity of model outputs to underlying distributions

For the results presented in the main text, we used log-normal distributions to capture the uncertainty in each of the model inputs. Figure 5 shows the sensitivity of the model output to individual parameters: Figure A.3 extends this analysis, repeating the parameter estimation using β distributions for all model inputs, as an alternative to log-normal distributions. The figure illustrates that this alternative choice of distribution does not substantially alter the model outputs, whether point or uncertainty estimates.

### Comparing infections from urban and rural tuberculosis cases

With the mean number of infections per year given as β and the mean duration of an infectious episode given by *D*, the number of infections arising from a single TB case (cumulated over the course of their disease) is the product β*D*. Figure A.4 gives β*D* values for rural, urban and national-level settings, showing that, although TB in rural settings can have a long duration, overall the cumulative *number of* secondary infections in urban settings tend to be higher than in rural settings.
